# Zinc and vitamin A supplementation fails to reduce sputum conversion time in severely malnourished pulmonary tuberculosis patients in Indonesia

**DOI:** 10.1186/1475-2891-9-41

**Published:** 2010-09-28

**Authors:** Trevino A Pakasi, Elvina Karyadi, Ni Made Desy Suratih, Michael Salean, Nining Darmawidjaja, Hans Bor, Koos van der Velden, Wil MV Dolmans, Jos WM van der Meer

**Affiliations:** 1 South East Asia Minister of Education Organization Tropical Medicine (SEAMEO TROPMED) Regional Center for Community Nutrition, University of Indonesia, Jl. Salemba Raya no.6, Jakarta Pusat 10420, Indonesia; 2 Department of Community Medicine Faculty of Medicine University of Indonesia, Jl. Pegangsaan Timur no.16, Jakarta Pusat 10302, Indonesia; 3 Department of General Medicine, WZ Johannes General Hospital Jl. Mohammad Hatta no.19, Kupang, Indonesia; 4 Department of Radiology WZ Johannes General Hospital, Jl. Mohammad Hatta no.19, Kupang, Indonesia; 5 Kefamenanu General Hospital, North Middle Timor District (TTU) Jl. Letjend Suprapto, Kefamenanu, Indonesia; 6 Department of Primary and Community Care, Radboud University Nijmegen Medical Center, Nijmegen International Center for Health Systems Research and Education, Geert Grooteplein Noord 21, Nijmegen 6525 EZ, the Netherlands; 7 Department of Internal Medicine, Radboud University Nijmegen Medical Center and Nijmegen Center for Infection, Inflammation and Immunity (N4I), Geert Grooteplein Zuid 8, Nijmegen 6525 GA, the Netherlands; 8 Micronutrient Initiative, Gedung Wirausaha Lt. 2 Jl. HR. Rasuna Said Kav C5, Jakarta 12920, Indonesia

## Abstract

**Background:**

A previous study showed that combination of zinc and vitamin A reduced sputum conversion time in pulmonary tuberculosis (TB) patients.

**Objective:**

We studied the efficacy of which single micronutrient contributed more to the sputum conversion time.

**Methods:**

In a double-blind randomized community trial, newly sputum smear positive pulmonary TB patients were assigned randomly to receive zinc, vitamin A, zinc + vitamin A or placebo on top of TB treatment. Patients were asked to deliver their sputum on weekly basis to measure positivity of the bacteria. Nutritional status, chest x-ray, hemoglobin, C-reactive protein (CRP), retinol and zinc level were examined prior to, after 2 and 6 months of treatment.

**Results:**

Initially, 300 patients were enrolled, and 255 finished the treatment. Most patients were severely malnourished (mean BMI 16.5 ± 2.2 Kg/m^2^). Patients in the zinc + vitamin A group showed earlier sputum conversion time (mean 1.9 weeks) compared with that in the other groups; however the difference was not significant. Also, no benefit could be demonstrated of any of the used supplementations on clinical, nutritional, chest x-ray, or laboratory findings.

**Conclusions:**

This study among severely malnourished TB patients, did not confirm that single or combined supplementation of zinc and vitamin A significantly reduced sputum conversion time or had other significant benefit.

## Background

The presence of micronutrient deficiencies among tuberculosis (TB) patients has led to the question whether micronutrient supplementation would give additional benefits for the patients on top of the TB treatment[1]. In a previous clinical trial of our group found that combination of zinc and vitamin A supplementation resulted in earlier sputum conversion than placebo, which began as early as 2 weeks after the administration of standard anti-TB treatment[2].

Vitamin A, as found as retinol in plasma, is one of important micronutrient which has specific immune function [3]. The presence of vitamin A deficiency in sputum-positive pulmonary TB patients compared with healthy subjects was confirmed[1,4]. and associated with the future of pulmonary adult TB patients [5].

Zinc is a trace mineral, which is essential for the function of cells of the immune system,[6] and a mild deficiency depresses the immune function in humans[7]. Zinc is also known as an essential mineral for normal mobilization of vitamin A from the liver to the plasma[8].

As in other programs for the combat against TB, the National Tuberculosis Program in Indonesia states that the disappearance of acid-fast bacilli (AFB) from sputum after treatment is basic in pulmonary TB patients' management. The presence of AFB in sputum can be assessed by direct visualization using the light microscope, and can be confirmed by growth of *Mycobacterium tuberculosis *in sputum culture. Conversion of sputum smears to AFB-negative status is only used in resource-limited countries[9] while in developed countries treatment success is measured by conversion to no growth of *Mycobacterium tuberculosis *(MTB) in sputum culture [10].

The effectiveness of anti-TB therapy is determined by several factors, including the burden of mycobacteria, underlying immune status, adherence to treatment, and drug susceptibility. In the face of new threats to combat tuberculosis, i.e. multidrug and extensively drug resistance MTB, and also immunosuppressive diseases (HIV, diabetes, malnutrition); the studies on micronutrients as modulator of the immune system are important. It would provide more evidence to the importance of supplementation against tuberculosis. Following the previous finding in Jakarta, the present study aimed to investigate the effect of single supplementation of zinc or vitamin A or the combinations, on the sputum conversion time and the health status of newly diagnosed pulmonary TB patients. The study was done in Nusa Tenggara Timur (NTT) province which was selected on purpose, because of its relatively poorer than in Jakarta with high prevalence of malnutrition and vulnerable of food insecurity as reported by the ministry of health in 2005.

## Methods

### Study design, time and location

The design of the study was a double-blind, randomized community trial. Prior to the study, randomization was done using a computer program, in which a treatment code was given to a subject. Every district had its random allocation table consisted 60 patients plus additional randomization for another 50 patients, to anticipate if one district had more patient than the other.

Patients were given standard treatment for TB and randomly divided into four supplementation groups: zinc alone, vitamin A alone, zinc + vitamin A, and placebo. The supplementation was taken daily and the patients were followed up until 6 months.

The primary outcome of the study was sputum conversion time. Secondary outcomes were: nutritional status (BMI, MUAC, % of body fat), abnormalities on chest x-ray, and results of blood examinations (CRP, plasma concentrations of zinc and vitamin A).

The study was conducted in Nusa Tenggara Timur (NTT) Province, Indonesia, covering four districts in Timor and Rote Island, namely Kupang City (the capital of NTT), Kupang District, Timor Tengah Utara (TTU-Northern Central Timor) district and Rote-Ndao district, from January 2004 until December 2005.

### Subjects and sample size

Subjects were newly diagnosed sputum smear-positive (SS+) TB patients aged 15-55 years. Prior and during the study, all pregnant or lactating females and subjects who had underlying chronic or degenerative disease, were excluded. All eligible patients were given information regarding the study including problems that might occur; and were asked to sign an informed consent for their participations in the study. The sample size was calculated based on the ability to determine a difference with α = 0.05 and 1-β = 0.80 in sputum conversion time, nutritional status (BMI) and plasma concentrations of retinol and zinc. As plasma retinol concentration was the parameter requiring the largest sample size, it was calculated that with a minimum sample size of 40 in each group, a between-group difference of 0.12 μmol/L in plasma retinol level could be detected based on the previous study.^2 ^Accounting for a 40% drop-out rate, each group in the intervention study comprised at least 56 subjects.

### Micronutrient supplementation and anti-TB drugs

Supplements and placebo were prepared by Kimia Farma Ltd, Indonesia, in the form of capsules. Each micronutrient capsule contained 1500 retinol equivalents (5000 IU) vitamin A (as retinyl acetate) and/or 15 mg zinc (as zinc sulfate) in a lactose matrix. Dosage of zinc was determined based on the recommended daily allowances for adult Indonesians. Based on the previous study in Jakarta, dosage of zinc was 15 mg in a form of zinc sulfate [2]. The vitamin A given in the capsule was twice of the Indonesian RDA, considering the possible deficiency and needs to overcome inflammation process. As found in the previous study in Jakarta, 5000 IU of vitamin A in combination with zinc, was considered adequate and used in the current study [2]. The placebo capsule consisted of lactose alone. All capsules were similar in terms of shape, color and size. Standard TB drugs were based on WHO guidelines, comprising 300 mg isoniazid, 450 mg rifampicin, 1500 mg pyrazinamide and 750 mg ethambutol daily for 2 months, followed by 600 mg isoniazid and 450 mg rifampicin three times a week during the next 4 months. To ensure adherence to the treatment, each patient had a treatment-partner as recommended by WHO in the DOTS strategy. The treatment-partner's task was to observe patients' compliance and to report whenever problems occurred regarding TB treatment. They got money when successfully maintained the treatment as it was given by the donor of this study. Compliance of the patients was measured monthly by the report of the treatment partner, double cross-checked with the blister of the TB drugs; as for the supplementation, we counted the left over capsules that should be taken every month.

### Data collection

All patients underwent physical examination, chest x-ray, nutritional and food intake assessment as described in other study [5], and blood analyses before the treatment started, after the intensive phase, and at the end of the treatment, except for sputum examination, which was done weekly. HIV testing was not carried out since the area is still known as a low prevalence area for HIV infection, as well as for HIV-TB co-infection[11].

#### Sputum conversion time

During the first 2 months of the study, the patients were asked to come to the clinic every week to deliver their sputum for direct AFB smear examination. Three early morning sputum specimens were taken from the patients and examined by direct microscopy after Ziehl-Neelsen staining in each Health Centre. The time was noted when the first of three weeks' consecutive sputum smears of good quality was negative. In the case that either, not all sputum samples were delivered, or the sputum was not of adequate quality, the data were considered missing.

#### Chest x-ray examination

Chest X-rays were performed at diagnosis on all patients and were evaluated by calculating the visible lesion area in both lungs. In patients with cavities, the total area of exposed cavity wall was calculated from the radius of visible cavities (πr^2^) as described before[2].

#### Nutritional status

Nutritional status was determined based on anthropometric measurements and micronutrient concentrations in the plasma. The anthropometrics measurements were: body weight, height, body mass index, skinfold thickness and percentage of body fat.

Body weight was assessed using an electronic platform model weighing scale (770 alpha; SECA, Hamburg, Germany) to the nearest 0.1 kg. Height was recorded to the nearest 0.1 cm using a microtoise. BMI was calculated as body weight divided by height squared (kg/m^2^). Mid upper arm circumference (MUAC) was measured using a flexible measurement tape. Skinfold thickness was measured at 4 sites: biceps, triceps, sub-scapular, and supra-iliac regions, with a slim guide skinfold callipers (Creative health product, Plymouth, Michigan, 48170), recorded to the nearest 0.2 mm. The percentage of total body fat was based on the skinfold data, and calculated using the Durnin and Wormesley equations[12].

#### Blood examination

Blood samples were collected between 08.00 and 10.00 AM in the local health center. Approximately 15 mL of fasting whole blood was withdrawn and separated into 4 vacutainers (Becton Dickinson, Rutherford, NJ) containing EDTA and heparin. Zinc and vitamin A concentration were measured in plasma. C-reactive protein (CRP) was measured to adjust the micronutrient deficiency in the statistical analysis. Plasma was separated after centrifugation at 750 × g for 10 minutes at room temperature, and then stored at minus 20°C until analyzed for CRP, retinol, and zinc concentration at the SEAMEO-TROPMED Laboratory, Jakarta. C-reactive protein (CRP) was measured using enzyme-linked immunosorbent assay (ELISA) method with a normal value < 10 mg/L. Plasma retinol concentration was measured by high performance liquid chromatography (HPLC), values less than 0.70 μmol/L were regarded as indicating deficiency, and values between 0.70 μmol/L and 1.4 μmol/L indicates marginal deficiency[13]. Plasma zinc was analyzed using atomic absorption spectrometry (AAS) with values >10.7 μmol/L regarded as normal. Determination of hemoglobin, WBC, and ESR were done on the same day at the provincial hospital laboratory. Hemoglobin and WBC were analyzed using automatic analyzer (ABX Micros 60, French). The cut-off points for normal hemoglobin were >120 g/L and >130 g/L for females and males respectively, and ESR was assessed using Westengren technique with a normal value < 20 mm/h. Serum albumin was measured using Spectrophotometer (Microlab 300, Merck, Germany) with a normal range 35-52 g/L.

### Statistical analysis

A one-sample Kolmogorov-Smirnov test was used to determine whether the variables were normally distributed. Data on the characteristics of the subjects at enrollment for their age and gender distribution, nutritional status, blood concentrations, and results of radiological signs were summarized and used to assess the comparability of the patients randomly assigned to the four treatment groups. Different means between groups were tested for significance using one-way ANOVA when normally distributed, and Kruskal-Wallis test when not-normally distributed. An extended analysis (ANCOVA) was further applied for determining predictors of outcome. Different proportions between groups were tested using chi-squared test. Within group changes were tested using paired student-t test for normally distributed data and the Wilcoxon signed-rank when for not-normally distributed. A p value less than 0.05 were considered significant. An intention to treat analysis was applied. Statistical analyses were performed using computer software SPSS for Windows PC version 14.0 (SPSS Inc, Chicago, IL, USA)[14].

### Ethical considerations

This study adhered to the Council for International Organizations of Medical Sciences guidelines (CIOMS, 1991). Data were collected after subjects agreed to participate in the study and gave written informed consent voluntarily. The research proposal was approved by the Ethics Committee of the Faculty of Medicine, University of Indonesia.

## Results

**Figure 1:**
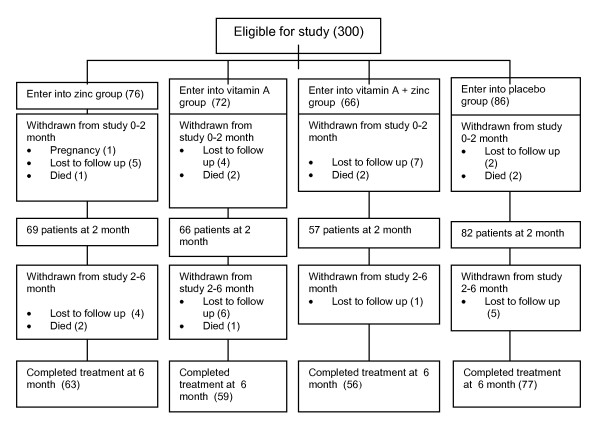
**Trial profile**. Including reasons for patients' drop out during the study.

A total of 300 patients were enrolled into the study and randomly grouped into 4 categories of intervention, i.e. supplementation of zinc (n = 76), vitamin A (n = 72), combined zinc + vitamin A (n = 66) and placebo (n = 86). After two months 274 patients were still in the study and analyzed, while 255 patients completed the study after 6 months and were analyzed. (Figure 1). Of all subjects, we received report from the treatment-partners that 22 patients (of total 255 patients who completed TB treatment) were not compliance to the study procedure.

Patients' mean age was 31.5 years and 63% of them were men (Table 1). Sputum positivity grade 3 was most prevalent. The prevalence of cavities on chest X-ray was almost 40% for all groups. These findings suggested that the patients had severe TB. There was no difference of baseline characteristics among the 4 groups of patients except for the proportion of patients with cavities (Table 1).

**Table 1:** Changes on sputum smear AFB and radiograph after 2 and 6 months

Variables	Groups			
	
	Zinc	Vitamin A	Vitamin A + Zinc	Placebo
Age (year), mean ± SD	30.9 ± 11.8	33.1 ± 11.5	30.1 ± 12	31.4 ± 10.4
Gender (n,%):				
Male	51 (67.1)	46 (63.9)	42 (63.6)	50 (58.1)
Female	25 (32.9)	26 (36.1)	24 (36.4)	36 (41.9)
Sputum smear grade (n,%):				
+ 1	18 (23.7)	22 (30.6)	25 (37.9)	24 (27.9)
+ 2	24 (31.6)	19 (26.4)	18 (27.3)	30 (34.9)
+ 3	34 (44.7)	31 (43)	23 (34.8)	32 (37.2)
Sputum conversion time (wk),mean ±SD	3.02 ± 2.2	2.1 ± 1.6	1.94 ± 1.4	2.5 ± 1.7
Duration to achieve WHO target(wk),mean †	6.6	4.0	4.0	5.0
Number of cavity (n,%)				
0 month*	18 (25.7)	35 (51.5)	25 (38.5)	36 (41.9)
2 month*	5 (7.5)	17 (27)	8 (14.8)	13 (17.1)
6 month	5 (8.1)	3 (5.3)	2 (3.6)	3 (4.2)
Cavity surface area (cm^2^), median (IQR)				
0 month	10.2 (4.9-17.4)	12.0 (6.2-22.1)	12.9 (4.8-15.9)	10.2 (4.9-21.6)
2 month	6.2 (2.5-21)‡	5.5 (2.5-13.7)‡	5.9 (2-16.2)‡	4 (3.1-10)‡
6 month	0 (0 - 0)‡	0 (0 - 0)‡	0 (0- 0)‡	0 (0 - 0)‡
Total lesion area (cm^2^), median (IQR)				
0 month	120 (71-179.8)	139 (44-216)	143 (73-198)	129 (62-209)
2 month	40.5 (12-100)‡	35 (0-80)‡	37 (9-94.5)‡	32.5 (4.5-77.5)‡
6 month	0 (0-42.4)‡	0 (0-25.8)‡	0 (0-21)‡	0 (0-25.8)‡

SD = standard deviation, IQR = interquartile range; *significant difference of proportion between groups (chi-squared, p < 0.05); significant changes from baseline p < 0.001‡; number of good quality sputum analyzed for zinc, vitamin A, zn + vitamin A and placebo groups were: 62, 57, 50, and 68 respectively (total 237). †WHO target: 85% subjects in a group converted into SS negative.

**Figure 2:**
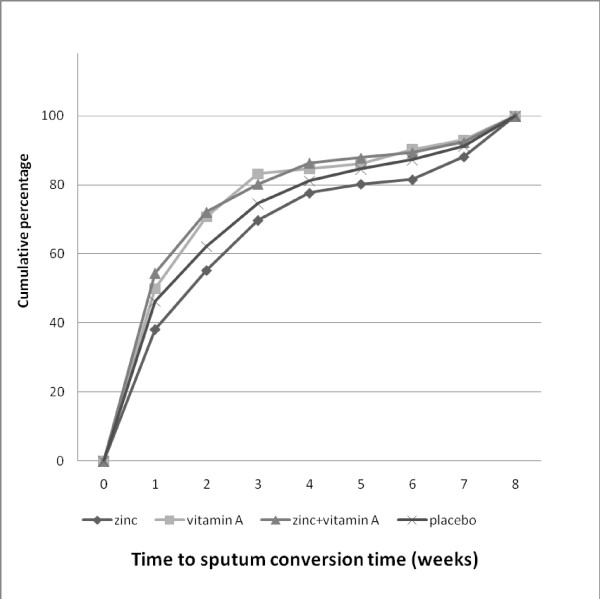
**Cumulative percentage of the sputum conversion time**. The zinc + vitamin A (n = 50) and vitamin A groups (n = 56) showed less weeks to reach 85% of sputum conversion, followed by the placebo group (n = 62).

In total 237 patients delivered all required sputum samples and of good quality and the rest was considered as missing data. As shown in table 1, mean sputum conversion time of the zinc + vitamin A intervention group was the shortest, and that of the zinc intervention group the longest. The zinc + vitamin A (n = 50) and vitamin A groups (n = 56) showed less weeks to reach 85% of sputum conversion, followed by the placebo group (n = 62). The zinc supplementation group (n = 68) showed the longest time to reach 85% of sputum conversion. However, no significant difference of the sputum conversion time was observed between groups (ANCOVA, p > 0.05). Factors included in the analysis were AFB level, compliance, and the differences of zinc and retinol level from the baseline. After one week, sputum was smear negative in 58.5% of patients from the zinc + vitamin A group, in 45.6% of patients in the vitamin A and placebo groups, whereas the zinc group showed the lowest percentage (35.3%). The zinc + vitamin A (n = 50) and vitamin A groups (n = 56) showed less weeks to reach 85% of sputum conversion, followed by the placebo group (n = 62). The zinc supplementation group (n = 68) showed the longest time to reach 85% of sputum conversion. After two months no significant differences were found between the groups (Figure 2). Taking into account the WHO success rate target of 85% conversion to negative, we observed that patients in the zinc + vitamin A supplementation group reached the target within 4 weeks, similar to the vitamin A group, followed by placebo group (5 weeks), and zinc group (6.6 weeks).

Mean BMI for the four groups varied from 16.4 - 16.6 and was 16.5 for all 300 patients, indicating severe malnutrition (Table 2). Fifty percent of the patients had BMI lower than 16.5 Kg/m^2 ^when enrolled in the study; the rest was moderate malnutrition (33%) and normal nutritional status (17%). The mean value of MUAC and percentage body fat were extremely low.

**Table 2:** Changes of anthropometric measurements prior to, at two and six months of treatment

Variables	Groups			
	
	Zinc	Vitamin A	Zinc+ Vit A	Placebo
BMI (kg/m^2^), mean ± SD				
0 month	16.5 ± 2.2	16.5 ± 2.2	16.6 ± 2.1	16.4 ± 2.5
2 month	17.7 ± 2.3‡	17.8 ± 2.2‡	17.7 ± 2.0‡	17.5 ± 2.4‡
6 month	18.5 ± 2.22‡	18.1 ± 2.42‡	18.3 ± 2.02‡	18.4 ± 2.62‡
Increasing BMI from the baseline				
(kg/m^2^), mean ± SD				
0 to 6 month	1.9 ± 1.3	1.7 ± 1.6	1.9 ± 1.5	2.0 ± 1.4
MUAC (cm), median (IQR)				
0 month	21.1 (19.8-23.1)	21 (20-23)	21.3(20-23.6)	21.8(19-23.6)
2 month	23 (21-25)‡	22 (20.1-24.7)‡	23 (21-24)‡	23 (20.8-24.5)‡
6 month	24 (22.5-25.1)‡	23.0 (21.8-25.0)‡	24.0 (22.0-25.3)‡	24 (22.5-26)‡
Body fat (%),mean ± SD				
0 month	11 ± 4.5	11 ± 6.2	10.8 ± 4.7	11.9 ± 5.9
2 month	12.9 ± 5.0‡	13.3 ± 5.9‡	12.9 ± 5.1‡	14.2 ± 6.1‡
6 month	14.5 ± 5.82‡	14.2 ± 6.42‡	14.2 ± 6.42‡	16.0 ± 6.32‡

SD = standard deviation, IQR = interquartile range, significant changes from baseline, paired student-T ‡p < 0.001

After 2 months of treatment, there were significant improvements of micronutrient status within groups compared with the initial levels, particularly in patients given combined vitamin A and zinc supplementation or placebo (Table 3). The proportion of cavities on chest x-ray was significantly different between the four groups but not after 6 months of treatment (Table 1). The changes of the proportions between groups however, were not significant. At the end of treatment, significant changes from the baseline were observed for all variables except for zinc concentration in the group receiving vitamin A and the group receiving zinc + vitamin A, but no difference between groups was observed (Table 1).

**Table 3:** Changes in blood parameters prior to, at two and six months of treatment

Variables	Groups			
	
	Zinc	Vitamin A	Zinc+ Vit A	Placebo
Plasma CRP (mg/L), median (IQR)				
0 month	25.7 (13.7-38.8)	27.9 (11.5-42.1)	29.1 (12.6-43.0)	30.9 (13.3-47.4)
2 month	4.6 (1.4-13.8) ‡	4 (1-14.3)‡	4.2 (1.3-15.5) ‡	6.4 (1.5-14.6) ‡
6 month	1.3 (0.5-2.7)‡	1.5(0.6-3.3)‡	1.1 (0.5-4.4) ‡	1.7 (0.9-40)‡
Serum albumin (g/dL), mean ± SD				
0 month	3.8 ± 0.84	4.0 ± 0.75	4.0 ±0.96	3.8 ± 0.83
2 month	4.5 ± 0.68‡	4.3 ± 0.88†	4.4 ± 0.74	4.4 ± 0.78‡
6 month	4.6 ± 0.52‡	4.6 ± 0.72‡	4.7 ± 0.62‡	4.6 ± 0.52‡
Leukocytes(cells/mm^3^),median (IQR)				
0 month	9300 (7575-13000)	9750 (7775-11575)	10200 (7475-12250)	9750 (7000-2225)
2 month	6700 (5400-8100)‡	6600 (5600-8050)‡	7500 (5500-8875)‡	7300 (5900-9100)‡
6 month	6000 (5100-7000)‡	6900(5275-8100)‡	6800 (5850-7800)‡	6800(5800-8100)‡
ESR (mm),median (IQR)				
0 month	73.0 (48.0-99.5)	59.5 (40.0-88.5)	62.0 (36.0-92.0)	75.0 (42.0-109.0)
2 month	28 (16-40)‡	31 (18-40)‡	30 (17.3-38.8)‡	28 (16-45)‡
6 month	11 (4-28)‡	14 (8-28.8)‡	12 (6-28)‡	11 (8-28)‡
Hemoglobin(g/dL), mean ± SD				
0 month	11.2 ± 2.7	11.6 ± 2.4	11.6 ± 2.5	11.0 ± 2.5
2 month	12.5 ± 2.78‡	12.6 ± 2.55‡	13.18 ± 2.48‡	12.12 ± 2.43‡
6 month	12.8 ± 2.3‡	13.0 ± 2.2‡	13.3 ± 2.2‡	12.8 ± 2.3‡
Plasma zinc(μmol/L), mean ± SD				
0 month	11.6 ± 2.2	11.9 ± 3	12.1 ± 3.0	11.8 ± 2.4
2 month	11.7 ± 2.43†	11.6 ± 1.83‡	12.5 ± 2.33‡	11.7 ± 2.33‡
6 month	13.1 ± 1.92†	12.5 ± 2.1†	12.8 ± 2.9†	12.6 ± 1.7 3‡
Plasma retinol(μmol/L), median (IQR)				
0 month	0.7(0.5-1.0)	0.7(0.5-1.5)	0.7(0.4-1.1)	0.7 (0.5-1)
2 month	1.2 (1-1.6)^a^‡	1.5(1-2)^ab^‡	1.3 (1-1.9)‡	1.2 (0.9-1.6)^b^‡
6 month	1.4 (1.1-1.9)†	1.6 (1.2-2.0)‡	1.6 (1.2-1.9)	1.4 (1.0-1.8)‡

SD = standard deviation, IQR = interquartile range, *significant difference of proportion between groups (chi-squared, p < 0.05), significant changes from baseline p < 0.05† and p < 0.001‡, ^ab ^significant changes between group (One-way ANOVA, p < 0.05)

## Discussion

In this double-blind randomized community trial, supplementation with zinc and vitamin A, either alone or combined, failed to show superiority over placebo. This was unexpected, as we could not confirm the results of the previous study in Jakarta, in which vitamin A and zinc supplementation was beneficial in terms of sputum conversion time[2]. Results similar to ours with regard to zinc supplementation were obtained in a study in Tanzania[15], where such supplementation did not lead to a reduction of sputum conversion time compared to supplementation with a multi-micronutrient or placebo either. Also, zinc supplementation did not improve immune response among TB patients infected with HIV in a study in Singapore[16]. The authors further concluded that in the absence of zinc deficiency, additional zinc supplementation was not beneficial. And to the best of our knowledge, the data on the effect of vitamin A supplementation in TB were inconclusive[17-19].

How can we explain the discrepancy between the results of the Jakarta study and the current results? The first explanation might be found in retinol concentrations and inflammatory response at baseline. In the Jakarta study, higher mean retinol concentrations (0.8 μmol/L and 0.9 μmol/L for the supplemented and placebo groups, respectively) were found than in the current study (0.7 μmol/L for all groups)[2]. The mean baseline CRP concentrations in the Jakarta study (53 mg/L for the micronutrient group and 44.1 for the placebo group) were almost twice as high as in the current study (mean CRP for all groups = 28.4 mg/L). Higher CRP concentrations reflect a stronger inflammatory response, which is known to lower the plasma retinol concentrations[20]. Thus, the patients in the Jakarta study may have had a lesser degree of vitamin A deficiency, inflammation leading to the low plasma retinol levels. This might imply that higher dosages of vitamin A supplementation would have been needed for the TB patients of the current study to reach retinol concentrations necessary for a clinical effect. This was also proven by the concentration of retinol at 2 and 6 months of intervention, which could not elevate the retinol level in the plasma. This is in line with the study in Malawi which concluded that supplementations at the level of recommended daily allowance (RDA) did not meet their main outcomes[21]. In contrast with this, the study in Tanzania which used multimicronutrient supplementations four to ten times higher than the RDA, reduced TB recurrences among the HIV-positive adults with TB, and increased T-cell counts of the HIV-negative patients[22]. With regard to zinc on the other hand, the level at baseline were already slightly above the deficiency cut off (10.7 μmol/L). This may explain the lack of efficacy observed for zinc[2].

The second explanation may be connected to the differences in the chest radiographs and the grading of sputum AFB positivity, an important predictor of sputum conversion[23]. The number of patients with cavities in our study was similar (38%) to that in the Jakarta study (37.5% of total patients in both group). More important however, is that in the present study, the proportion of patients with cavities among the four groups differed significantly at baseline (Table 1), with the highest proportion of cavities (51.5%) being present in the patients of the vitamin A supplementation group. This imbalance may have obscured the effect of vitamin A supplementation but would not explain the lack of effect in the zinc + vitamin A group. Findings of a Spanish study showed the association between the presence of cavity and the conversion time in the presence of HIV co-infection. It was concluded that cavity prolonged the conversion time, and the presence of HIV lowered the prevalence of cavity production in the lungs as captured in the chest x-ray. Thus, the absence (probably very small proportion) of HIV co-infection in the current study led to cavity formation in the lung and at the end prolonged the conversion time [24].

The severity of TB in our patients is witnessed by the fact that 40% had sputum AFB positivity grade 3 prior to treatment whereas in the Jakarta study 68% had grade 1 (for both supplemented and placebo groups) and this can be expected to lead to longer sputum conversion time[23]. It may be that the supplementation effect does not become clear in such severe cases.

The third explanation for the difference between the current study and the previous study in Jakarta is related to the sample size. In Jakarta, the sample size was estimated "on the ability to determine a difference of *retinol cosentration in plasma*, with an alpha = 0.05 and 1-beta = 0.95 with use of a one-tailed test for concentrations of hemoglobin in blood, and of retinol and zinc in plasma."[2] The findings in the Jakarta trial, of an effect of zinc+vitamin A supplementation on the sputum conversion time was unexpected as no sample size was estimated for this specific outcome. In the present study, designed for confirmation of the previous results, the effect on sputum conversion time was not found. Thus, the probability is real that the finding in the first study was not valid, due to underpowerment of the study to measure a difference in sputum conversion time.

Another explanation may be related to the observation that the patients in the current study were more malnourished than the patients in the Jakarta study. Mean BMI of our cohort was 16.5 Kg/m^2 ^as compared to 18.5 Kg/m^2 ^in the Jakarta study[2]. There was no available population based study on adult malnutrition, however a recent study found 33% of under nutrition occurred among neighbors of the TB patients[25]. Such a low BMI may reflect two processes. One would be protein energy malnutrition (which severely affects host defense) and the other wasting due to the catabolism induced by the acute phase response[26]. As found in our subjects, the BMI increased along with reducing of inflammation. When the patients got healed, as shown in the total lesion area of lung, they gained more weight and also of other micronutrients, such as iron, zinc and vitamin A. (table 1 and 3) This might explain how the hemoglobin increased, although it was still in the borderline to be considered normal.

Neither of low intake or wasting is directly affected by supplementation of micronutrients and hence it may not be remarkable that we failed to show an effect in this study. Beside macronutrient deficiencies, there was also the possibility that additional micronutrients deficiency were present in the study population, which could not be corrected with zinc and vitamin A.

For example, vitamin D has been implicated in defense against TB, but vitamin D deficiency is a worldwide problem. Furthermore there might be a link between vitamin D deficiency with ethnicity. In the Jakarta study, the patients were mainly from Java, Sunda and Sumatra, with a minority of others, the current study consists mainly of patients indigenous to Timor and Rote islands[27]. We found in the same study areas that ethnicity was associated with the development of TB and severity of TB[28,29]. In line with this, a recent study showed that vitamin D receptor genetic polymorphisms were associated with the time to sputum culture conversion[30]. Also it was shown that vitamin D receptor genotype independently predicted the sputum smear conversion time while on anti-TB therapy[31]. One might speculate that ethnic background also plays a role in the response to supplementation with micronutrients.

We conclude that the patient groups studied here, were suffering from severe tuberculosis with cavities in at least one third of the patients, and high sputum positivity grade. The patients were vitamin A deficiency whereas zinc was at borderline but sufficient to support the immune system and perhaps more important, also severely malnourished. It was also important to notice that the supplementation could not elevate the level of retinol and zinc plasma higher than its borderline. Against this background we were not able to replicate the results of the Jakarta study that demonstrated a beneficial effect of vitamin A and zinc in tuberculosis. This means that the previous findings in Jakarta cannot be generalized. For further studies, a larger sample size will be required, and higher dosages of the zinc and vitamin A supplementations should be considered. Also, in patients with very low BMI, the effect of protein supplementations needs further study.

## List of Abbreviation

AAS: (atomic absorption spectrometry); AFB: (acid fast bacilli); ANOVA(Analysis of variance); ANCOVA: (Analysis of covariance); BMI(body mass index); CIOMS: (Council for International Organization of Medical Sciences); CRP(C reactive protein); ELISA: (enzyme-linked immunosorbent assay); ESR: (erythrocyte sedimentation rate); HIV(Human immunodeficiency virus); HPLV: (high performance liquid chromatography); MUAC(mid upper-arm circumference); NTT: (East Nusa Tenggara Province); RDA(Recommended daily allowance); SPSS: (Statistical Package for Social Science); SS(sputum smear); TB: (tuberculosis); TTU: (Northern Central Timor District); WBC: (white blood cell); WHO: (World Health Organization)

## Competing interests

We declared that there is no competing interest, neither financial aspect nor other aspects, in pursuing and publish the research.

## Authors' contributions

TAP lead the data collection in the field, did the analysis and writing up the manuscript. EK was the primary investigator who designed the study. NMDS, MS and ND gave contribution in patient management and monitoring. HB helped in the statistical analysis. KvdV, WMVD, and JvdM helped in supervising the analysis, assessment of the findings, and writing up the whole trial. All authors have read and approved of the final version of the manuscript.

## Authors' information

The study was the continuation of the previous one published in Am J Clin Nutr 2002;75:720-7 when EK got her promotion from the Radboud University Nijmegen Medical Center, under the supervision of JvdM. The first author was a candidate doctor under the supervisions of JvdM, KvdV and WMVD.
